# Repositioning the Canadian CKD Clinic Network Through Structured Input and World Café Dialogue: A Conference Report

**DOI:** 10.1177/20543581261470157

**Published:** 2026-07-17

**Authors:** Selina Allu, Mila Tang, Monica C. Beaulieu, Aminu K. Bello, Micheli Bevilacqua, Helen Hoi-Lun Chiu, Kate Chong, Maoliosa Donald, Mark D Elliott, Meghan J Elliott, Manuel Escoto, Isabella Ethier, Janet Graham, Adeera Levin, Judith G Marin, Kelly Picard, Steven D Soroka, Christine A White, Cathy Woods, Tae Won Yi, Matthew T. James

**Affiliations:** 1 Can-SOLVE CKD, Vancouver, BC, Canada; 2 Division of Nephrology, Cumming School of Medicine, 2129 University of Calgary, Calgary, AB, Canada; 3 Health Research BC, Vancouver, BC, Canada; 4 Division of Nephrology, 8166 University of British Columbia, Vancouver, BC, Canada; 5 Department of Medicine, 3158 University of Alberta, Edmonton, AB, Canada; 6 BC Renal, Vancouver, BC, Canada; 7 The Kidney Foundation of Canada- BC & Yukon Branch, Burnaby, BC, Canada; 8 Department of Medicine, Department of Community Health Sciences, 2129 University of Calgary, Calgary, AB, Canada; 9 Division of Nephrology, Department of Medicine, 25443 Centre Hospitalier de l’Université de Montréal, Montréal, QC, Canada; 10 Health Innovation and Evaluation Hub, Centre de Recherche du Centre Hospitalier de l’Université de Montréal, Montréal, QC, Canada; 11 Faculty of Medicine, Université de Montréal, Montreal, QC, Canada; 12 27337 The Ottawa Hospital, Ottawa, ON, Canada; 13 102794 Providence Health, Vancouver, BC, Canada; 14 Pharmaceutical Sciences, 8166 University of British Columbia, Vancouver, BC, Canada; 15 Island Health Authority, BC Renal, University of British Columbia, Nanaimo, BC, Canada; 16 Nova Scotia Health Authority, Halifax, NS, Canada; 17 Division of Nephrology, Dalhousie University, Halifax, NS, Canada; 18 Department of Medicine, Queen’s University, Kingston, ON, Canada; 19 Division of Nephrology, University Health Network, Toronto General Hospital, University of Toronto, Toronto, ON, Canada

**Keywords:** chronic kidney disease, learning health system, participatory engagement, priority setting, CKD clinic network

## Abstract

**Purpose of program:**

This conference report describes findings from activities to reposition the Canadian Chronic Kidney Disease (CKD) Clinic Network by identifying research priorities and actionable steps aligned with the 2024 Kidney Disease: Improving Global Outcomes (KDIGO) guidelines. The initiative aimed to identify priority actions to integrate clinical research and care through collaborative dialogue and community-driven planning.

**Sources of information:**

The 2024 KDIGO Clinical Practice Guideline for CKD served as the foundation to assess and prioritize relevant research areas. Insights were gathered through a national survey and a participatory workshop using World Café methodology.

**Methods:**

An anonymized online semi-structured survey was distributed to members of the Canadian CKD Clinic Network from April 4-14, 2025 to assess the perceived relevance and importance of the 2024 KDIGO CKD guideline research recommendations. This was followed by an in-person World Café workshop in May 2025 involving patients, clinicians, researchers, and administrators. Data collection included live graphic recording, table notes, and Post-it reflections, which were thematically analyzed.

**Key findings:**

The survey yielded responses from 80 network members, representing a 24% response rate. Survey results prioritized the need for research on: the impact of newer medications (SGLT2i, non-steroidal mineralocorticoid receptor antagonists) in patients intolerant of ACEi/ARB, implementation science to ensure uptake of proven therapies and symptom management, essential components for transition clinics for young people, tools for health literacy in different populations, strategies to prevent hyperkalemia, impact of medication deprescribing, effects of dietary restriction, and symptom identification, classification, and control. Attendees of the World Café (n=19) identified several network strengths, including its established relationships that drive national collaboration, willingness in knowledge sharing, and inclusion of the patient voice. Feedback emphasized the need for collaborative implementation and context-sensitive application of guidelines. The workshop also aligned the network’s future direction with Learning Health System principles, which embed research into routine care delivery, supporting goals with provincial and national CKD strategies, deepening patient engagement, shared measurement tools, and local-to-national learning loops. Actionable steps identified included enhancing visibility through a public-facing website, anchoring activities to sustainable national platforms, and exploring additional partnerships and collaborative research opportunities.

**Limitations:**

Only 24% of the CKD Clinic Network responded to the survey, and 19 members were present at the World Café, reflecting perspectives from only a small portion of the network. Therefore, the results may overlook key challenges or opportunities that did not surface due to missing perspectives of others within the kidney care community.

**Implications:**

This initiative demonstrates how engaging diverse perspectives can inform network transformation and future strategic research directions. The CKD Clinic Network is well-positioned to evolve into a responsive, patient-centred platform that drives learning and improvement in health care delivery across Canada’s kidney care system.

## Purpose of the Program

Chronic kidney disease (CKD) management in Canada is challenged by low public awareness, fragmented and inconsistent clinic models, workforce and resource constraints, inequitable access to care and medications, limited patient education, weak data systems, and gaps between evidence and practice.^1-8^ Enhancing national care models, strengthening multidisciplinary teams, improving access to therapies, standardizing data collection, and improving patient-centred education and decision-making could improve the quality of care and patient outcomes. A coordinated national CKD clinic network might play a central role by harmonizing care pathways, expanding best practices, integrating with primary care, tracking outcomes, and embedding continuous quality improvement to deliver more equitable, consistent, and effective care across the country.

The Canadian Chronic Kidney Disease (CKD) Clinic Network was originally established in 2013^9^ as part of the Timing of Dialysis Initiation project under the Canadian Kidney Knowledge Translation and Generation Network (CANN-NET) to support evidence-based clinical practice across multidisciplinary kidney care clinics in Canada.^10^ These Kidney Care Clinics play a central role in coordinating care for people with CKD^11^ given evidence that variations in CKD care quality are associated with important differences in patient outcomes.^4^ Initially composed of frontline kidney care providers, the Network has since evolved into a broad, inclusive community of patients, caregivers, clinicians, researchers and research team members, administrators, and decision-makers.

The overarching purpose of the CKD Clinic Network is to promote equitable, high-quality CKD care by fostering collaboration, sharing innovations, and enabling the integration of research into clinical practice. Since 2017, the Network has hosted quarterly webinars to showcase leading practices, share emerging evidence, and strengthen national connections, functioning as a knowledge mobilization arm of the Canadians Seeking Solutions and Innovations to Overcome Chronic Kidney Disease (Can-SOLVE CKD) Network.^12^

In 2024, recognizing a rapidly evolving landscape marked by the release of updated Kidney Disease: Improving Global Outcomes (KDIGO) Clinical Practice Guideline (CPG) for the Evaluation and Management of CKD,^13^ new therapeutic options, persistent variability in clinic models, and a growing emphasis on integrated, patient-centred care, the CKD Clinic Network initiated a renewal process to reflect on its current role and future direction.

Rather than defining a fixed set of deliverables, this renewal process aimed to open space for reflection, honest dialogue, and collaborative planning. Aspirational directions that emerged throughout this process included: (1) aligning with updated 2024 CKD KDIGO CPG and integrating emerging therapies; (2) addressing variation in care delivery and funding models across provinces and territories^14^; (3) enhancing equitable patient access to research and therapies; (4) strengthening the Network’s role as a trusted knowledge intermediary within the Canadian kidney research ecosystem; and (5) building a sustainable, inclusive, and visible national platform to support research collaboration, innovation, and policy advancement in CKD care.

This report describes the first phase of this renewal effort, including the methods and reflections from a national survey undertaken in April 2025 and an in-person World Café workshop held in May 2025. It highlights shared research priorities, themes for network evolution, and actionable next steps identified by diverse participants. Together, these insights provide a foundation for repositioning the CKD Clinic Network as a responsive, learning-oriented, and patient-centred platform for the future of kidney care in Canada.

### Sources of Information

The 2024 KDIGO CPG for Chronic Kidney Disease (CKD) served as the foundational framework for both phases of this initiative — a national survey and a subsequent in-person World Café workshop. These guidelines provided a shared evidence base to assess the relevance, applicability, and gaps in current research and practice across Canadian CKD clinics.

To explore the perspectives of the Canadian CKD Clinic Network on these guidelines and their implementation potential, an anonymized national survey was developed and disseminated online in April 2025. The survey received 80 responses (∼24% response rate of the membership of the CKD Clinic Network), representing a broad cross-section of non-dialysis nephrology programs from 10 provinces. The survey invited respondents to reflect on selected KDIGO CKD research recommendations and share their perspectives on what areas were most relevant, needed, or actionable within their own clinical or regional settings. Respondents included clinicians, program administrators, and members of multidisciplinary kidney care teams, offering insights into how guideline recommendations intersect with frontline realities.

Building on survey findings, a one-day in-person World Café workshop was held in Vancouver in May 2025. Nineteen participants — including patient partners, nephrologists, nurses, administrators, and researchers — gathered to share what matters most to them in advancing CKD care in Canada. Rather than ranking or scoring predefined options, the session invited open dialogue, collective reflection, and idea generation. This enabled the network to surface key themes, opportunities, and shared aspirations to inform its future direction.

## Methods

This initiative involved two complementary activities: a national online survey conducted in April 2025 and an in-person World Café workshop held in May 2025. Additional materials supporting these activities, including the survey instrument, consent form, detailed World Café report, application of design principles, full survey results, and participant list, are available as Supplementary Materials.

## Online CKD Clinic Network Members Survey (April 2025)

In April 2025, a national survey was conducted to assess the perceived relevance and importance of research topics outlined in the 2024 KDIGO CPG for CKD.

### Participants and Recruitment

The survey was pilot-tested by co-authors MD and MJ. The survey was presented using Qualtrics XM (Qualtrics, Provo, UT)^15^ and distributed via email to 326 members of the Canadian CKD Clinic Network, comprising nephrologists, nurses, allied health professionals, researchers, administrators, patients, and others involved in non-dialysis multidisciplinary kidney care programs across Canada. Ethics approval was obtained from the University of Calgary Conjoint Health Research Ethics Board (REB25-0365). Invitations were distributed via email to the network mailing list, with one reminder sent during the survey period. The survey was open for 10 days, from April 4 to April 14, 2025. Participation was voluntary and anonymized.

### Data Collection

The survey explored perspectives related to the KDIGO CKD CPG research recommendation statements across key domains including health literacy, nutrition, symptoms, and emerging therapies. Respondents were asked to rate the importance of each recommendation using a five-point Likert scale (ranging from “not important” to “very important”), or to indicate “prefer not to answer.” Optional open-text questions invited additional input on gaps, needs, or emerging priorities.

The survey consisted of 23 questions, including Likert-style ratings and open-text fields. Respondents were asked to assess the perceived relevance and importance of selected research-related recommendations drawn from the 2024 KDIGO CPG for CKD. The survey focused on the three KDIGO CPG chapters most relevant to CKD Clinic Network activities: (1) Chapter 3: “Delaying CKD progression and managing its complications”, (2) Chapter 4: “Medication management and drug stewardship in CKD”, and (3) Chapter 5: “Optimal models of care” (Supplementary Material 1).

### Data Analysis

Responses were collected anonymously. Likert scale responses were summarized descriptively using stack bar charts to visualize the number of responses in each category. Responses to open-ended questions were analyzed thematically to identify recurring topics and insights related to guideline implementation, research priorities, and system-level needs. The highest-ranked research topics were determined based on the percentage of respondents who rated each item as “very important” (score of 5 on a 5-point Likert scale). Survey findings were subsequently presented to members of the CKD Clinic Network in a one-hour webinar on April 17, 2025, providing an opportunity for clarification, discussion, and reflection prior to the World Café session.

## In-Person World Café (May 6, 2025)

### Participants and Recruitment

Following the presentation of the survey results, a one-day in-person World Café workshop was held on May 6, 2025, in Vancouver, British Columbia. The World Café format^16^ was selected to support our objective of surfacing shared values, aspirations, and potential directions through peer-to-peer knowledge exchange. Nineteen participants attended, including patient partners, nephrologists, allied health care practitioners, researchers, administrators, and representatives from relevant organizations. Participants were recruited through a targeted invitation process. Individuals had previously self-identified as interested in contributing to the CKD Clinic Network refresh during the development of a Canadian Institutes of Health Research (CIHR) Planning and Dissemination Grant proposal. Efforts were made to ensure balanced representation across disciplines involved in CKD clinic care (including clinicians, administrators, researchers, and patient partners) as well as geographic diversity from major programs and provinces across Canada. Participants provided informed consent in advance.

### Data Collection

The three-hour session followed the World Café method,^17^ which emphasizes inclusive dialogue, cross-pollination of ideas through rotating rounds of questions, and collaborative sense-making. Participants were seated at small café-style tables of four and engaged in two rounds of conversations in response to pre-determined questions about the future of the CKD Clinic Network. Table discussions were captured through a combination of (1) participant-generated Post-it notes, (2) individual table notes, (3) a live recording by a professional graphic illustrator, and (4) an “Idea Wall” where key themes were synthesized in real time.

### Data Analysis

After the workshop, the table notes, Post-it notes on the “Idea Wall”, and the graphic illustration were consolidated. Thematic analysis was conducted by two members of the research team (SA and MT), both experienced in qualitative methods and involved in co-facilitating the World Café session. An inductive approach was used, allowing themes to emerge from the data (including sticky notes, table notes, and facilitator reflections) rather than applying a predefined framework. Discrepancies were resolved through discussion, with additional input from the World Café convener (MJ) and an implementation science expert participant (MD) to enhance credibility and interpretation. This iterative thematic analysis generated a set of “idea clusters,” actionable steps, and shared priorities, which informed the development of a post-event report (Supplementary Material 2) and the foundations of a renewed CKD Clinic Network strategy.

### World Café Session Activities

(a) Welcome, Context Setting, and Participant Orientation

The World Café session was convened by a nephrologist (MJ) with national leadership in nephrology research and co-hosted by two knowledge translation practitioners (SA and MT) with expertise in participatory methods and implementation support. The co-hosts welcomed participants, introduced the purpose of the gathering, and grounded the group in shared expectations and a sense of collective purpose.

(b) Applying World Café Principles and Guidelines

The session was intentionally designed using the World Café’s seven design principles^17^ and etiquette guidelines to support meaningful, respectful, and inclusive dialogue.^18^ These principles informed all aspects of the session—from question design and room layout to timing and tone—and were introduced at the outset to set the tone for generative conversation. A summary of how these principles were applied is included in Supplementary Material 3.

(c) Two Rounds of Dialogue and Table Mini-Harvests

Participants engaged in two rounds of facilitated discussion based on generative prompts: (1) identifying the unique strengths and value the CKD Clinic Network offers to research, and (2) imagining bold steps toward evolving into a more responsive research platform. There were 3–4 participants per table with intentionally diverse groupings (e.g., mixing patient partners, clinicians, and administrators) to foster cross-pollination of ideas. Group insights were shared during brief mini-harvests (at the end of each round of discussion) and breaks between rounds encouraged informal exchange.

(d) Visual Reflection and Gallery Walk

Following the table rounds, a live graphic recorder shared preliminary illustrations summarizing key themes (the “Illustration Wall”). Participants then engaged in a gallery walk to explore the “Illustration Wall” and “Idea Wall,” reviewing Post-it notes and visuals, clustering related concepts, and adding new reflections to further co-develop emergent themes.

(e) Full Group Harvest and Collective Closure

The session closed with a reflective “head–heart–hands” sharing circle, where each participant contributed insights using a symbolic web of yarn to visualize their connection to the group and to the work ahead. This collective harvest allowed for deeper integration of ideas, and the facilitators concluded by thanking participants and outlining next steps for the CKD Clinic Network.

## Key Findings

### A. Online Survey

#### Survey Respondent Demographics

A total of 80 respondents from across Canada participated in the survey. The majority were based in Ontario (n=25), British Columbia (n=17), and Alberta (n=13), with additional representation from seven other provinces. Most respondents reported working in large urban centres, with 60% (n=48) residing in cities with populations of 500,000 and over.

The largest group of respondents were nephrologists (n=37), followed by individuals who identified as managers, decision-makers, researchers, or patient partners (n=22), and CKD clinic nurses (n=9). Although the survey questions focused primarily on clinical and operational perspectives, three patient partners also completed the survey, contributing lived-experience insights to the findings. Respondents had significant clinical experience, with more than half (n=42) having worked in their current clinical roles for over 10 years.

In terms of demographic diversity, the majority identified as female at birth (n=55), and most identified as cisgender women (n=47). Age distribution skewed toward mid to late career professionals, with 68% between the ages of 41–60. Most respondents identified as White (n=55), with smaller numbers identifying as Asian (South, East, and Southeast), Indigenous, or other backgrounds.

#### Survey Completion and Response Patterns

Of the 80 individuals who initiated the survey, not all respondents completed every question. A pattern of progressive attrition was observed, with response rates decreasing across subsequent sections, with 52 completing the final section. As such, the number of responses per item varied, and all percentages reported are based on the number of respondents who completed each respective question.

#### Key Findings From Prioritized Research Topics

The responses to all questions are displayed in stacked bar plots provided in Supplementary Material 4. Response rates varied by section; thus all percentages of response rates are based on the denominator of respondents who completed each respective question.

The top 9 highest-ranked research topics, based on the percentage of respondents who rated each item as “very important” (score of 5 on a 5-point Likert scale), are shown in Table 1. Topics were ranked according to the proportion of respondents assigning this rating. The nine topics presented each had more than 30% of respondents rating them as “very important,” whereas subsequent items did not meet this level of endorsement.

**Table 1.** Highest-Ranked Research Topics

KDIGO CKD research recommendation	Number rating topic as very important/Total number of responses (%)*
Impact of newer agents (SGLT2i, nsMRAs) in patients intolerant to ACEi/ARB (Chapter 4.9)	23/52 (44.2)
Implementation science to ensure uptake of proven therapies and symptom management (Chapter 5.6)	20/53 (37.7)
Essential components of transition clinics (for positive outcomes on young people with CKD) (Chapter 5.7)	19/53 (35.8)
Develop reliable tools for health literacy for use in different populations (Chapter 5.2)	19/53 (35.8)
Investigate best strategies to prevent hyperkalemia (Chapter 3.19)	19/59 (32.2)
Impact of deprescribing medications (Chapter 4.8)	17/53 (32.0)
Effect of dietary restriction on quality of life (Chapter 3.4)	19/60 (31.7)
Platform studies to evaluate common symptoms (Chapter 5.5)	16/52 (30.8)
Identification and classification of common symptoms (qualitative and quantitative methods) (Chapter 5.4)	16/53 (30.1)

*Note. Percentages reflect the proportion of respondents who rated the item as “very important” (score of 5 on a 5-point Likert scale).

Highly prioritized research topics included the impact of newer agents (SGLT2i, non-steroidal mineralocorticoid receptor antagonists) in patients intolerant of ACEi/ARB, with 44% indicating this as very important. Other highly ranked items included developing reliable tools for health literacy in different populations (35.8%) and transition clinic components to support young people with CKD (35.8%). Implementation-related research was also a key priority, with 37.7% rating the importance of implementation science to ensure uptake of proven therapies and symptom management. Additional highly rated topics included preventing hyperkalemia, deprescribing medications, and improving the identification, classification, and management of common CKD-related symptoms.

## B. In-Person World Café Session

### World Café Participant Demographics

Nineteen individuals participated in the in-person World Café workshop. Participants represented a diverse cross-section of the kidney care community, including 2 patient partners, 10 nephrologists, 2 allied health care professionals, 2 organization representatives, 2 researchers, and 1 renal program administrator. This diverse group was intentionally assembled to represent a range of individuals including those living with CKD, clinical perspectives, and system-level insights. Participants were drawn from across Canada, offering a national lens on shared challenges, opportunities, and bold ideas for strengthening the Canadian CKD Clinic Network.

**Text Box 1. Participant reflection on the World Café experience**

“*We got so much accomplished in a short period of time, and it was surprisingly easy to make it happen. I would highly recommend the World Café for any brainstorming session where diverse perspectives and quick results are needed. What we accomplished in three hours was remarkable. Having the right people in the room, in an intimate setting, created an environment where meaningful discussion and collaboration flourished. It truly showed the power of bringing the right people together at the right time, all focused on developing an action plan and solutions to move forward. I remain committed to the process that worked so well for us. It was an honour to participate*.”

Participants came from six provinces across Canada: British Columbia, Alberta, Ontario, Quebec, Manitoba, and Nova Scotia, providing a broad geographic representation spanning Western, Central, and Atlantic regions.

Reflections from participants underscored the value of bringing diverse stakeholders together in an intimate, structured setting to accelerate shared understanding and action planning (Text Box 1).

### World Café Conversations and Key Findings

**Figure 1.**
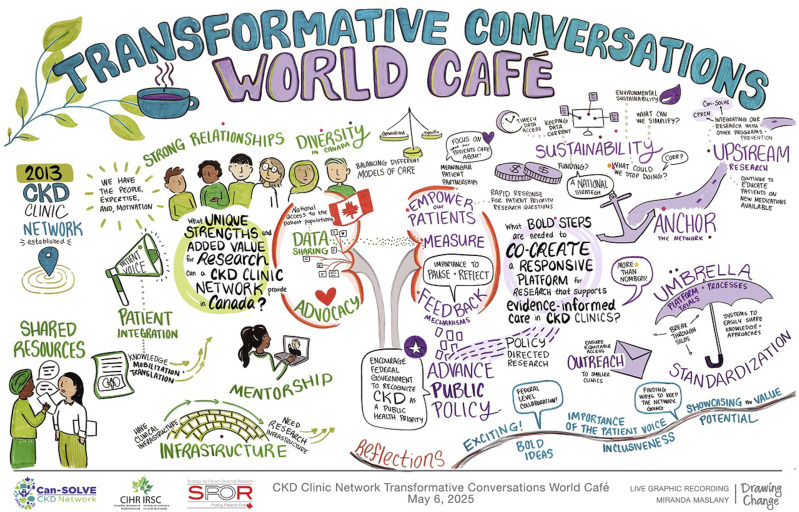
Transformative conversations world café graphic illustration

The World Café workshop uncovered bold ideas for enhancing the network’s impact and explored its evolution into a more responsive and sustainable research platform. This section summarizes key themes that emerged across the two rounds of dialogue and the final full group harvest. The discussion below builds on the overall executive summary captured in the graphic illustration below (Figure 1) and the detailed report (Supplementary Material 2).

1. Network Strengths: A Foundation to Build Upon

Participants affirmed the value of the CKD Clinic Network as a collaborative space that bridges silos across research, clinical care, and administration. The network was seen as an effective mechanism to foster a sense of community, promote shared learning, and advance national conversations on kidney care. Several core strengths were highlighted, including its capacity to bring together diverse perspectives, support patient engagement in research, and align quality improvement efforts across jurisdictions. Participants also appreciated the network’s evolving identity as a connector and enabler of implementation by providing opportunities to test ideas, share resources, and build synergy across teams.

2. Actionable Steps and Emerging Opportunities

Looking ahead, participants articulated a shared desire to see the CKD Clinic Network evolve into a more dynamic and action-oriented platform. One idea was to enhance the network’s role as a community of practice, where ongoing peer-to-peer exchange, mentorship, and joint problem-solving could flourish. There was also a strong appetite to embed research more directly into care delivery, using the network as a space to co-design and test innovations that are clinically meaningful and practically feasible. This included the aspiration to support teams in building research-care integration, particularly through quality improvement initiatives, feedback loops, and shared implementation tools. Many participants emphasized that this would require a stronger voice for patients and caregivers, calling for more intentional and consistent roles for lived experience partners in setting priorities, shaping research, and guiding implementation.

Another emerging, though ambitious opportunity involved enhancing the Network’s capacity to inform broader system transformation. This included standardizing and strengthening data systems across clinics to enable benchmarking and shared learning, as well as growing the network’s ability to influence health policy through coordinated advocacy and mobilizing knowledge. The collective sense was that these steps would enable the network to function not only as a research dissemination platform but as a responsive, equitable, and learning-driven engine for kidney care policy improvement in Canada.

**Figure 2.**
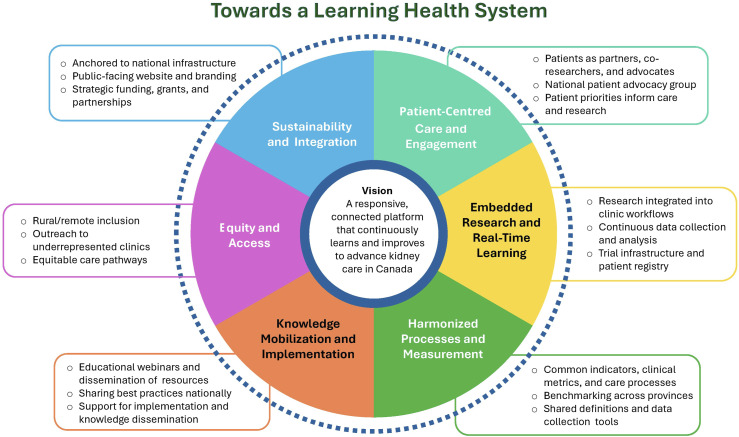
Reimagined CKD network: Supporting learning health systems

The discussions strongly aligned with Learning Health System (LHS) principles^18^ (details in the World Café Report Supplementary Material 3), which emphasize embedding research into routine care delivery, as well as the importance of continuous improvement, real-time feedback, and meaningful patient and provider involvement. Participants envisioned a future where the CKD Clinic Network could serve as a learning-oriented, policy/advocacy-enabled, and implementation-focused platform. This would involve building feedback loops between evidence, practice, and policy, and creating an environment where learning is embedded into the structure and culture of kidney care (Figure 2).

### Recommendations

World Café participants emphasized the need to continue fostering dialogue across the CKD Clinic Network to maintain momentum and coalesce around shared priorities. There was strong interest in using the network as a springboard to co-develop a national kidney research and quality improvement platform—one that is grounded in real-world clinical experience and responsive to national priorities and local needs.

Participants identified a set of priority recommendations to guide the next phase of the CKD Clinic Network’s development. These recommendations, synthesized from workshop discussions and the post-event report, are summarized in Table 2.

**Table 2.** Priority Recommendations for the Future Development of the Canadian CKD Clinic Network

No.	Priority recommendation
1	Enhance the CKD Clinic Network as a national community of practice that embodies the principles of a Learning Health System
2	Align network goals with provincial and national CKD strategies and health system improvement efforts
3	Deepen patient engagement by supporting meaningful roles for patient partners in co-designing projects and participating in network leadership and governance
4	Invest in data collection and shared measurement tools to enable benchmarking, quality improvement, and evidence-informed decision-making
5	Support local-to-national learning loops by facilitating storytelling, case studies, and implementation learning exchanges
6	Map network capacity and infrastructure needs to identify resources required for sustainability and growth (e.g., personnel, coordination capacity, funding, tools, and time)
7	Secure funding and governance structures that support sustainable growth and adaptability of the network

A recurring recommendation was the development of practical supports to enable guideline implementation and knowledge mobilization, particularly tools that reflect patient perspectives and contextual variation across clinics. Several participants called for a process to revisit and update priorities as the landscape of kidney care evolves, including clearer pathways to action, stronger linkages with health system leaders, and visible champions across regions. Suggestions also included increasing visibility of the network to funders and decision-makers, leveraging the network’s pan-Canadian reach to identify scalable innovations, and creating more inclusive spaces for sustained patient involvement.

Together, these recommendations emphasize the need for sustained coordination, enhanced visibility, stronger partnerships, and mechanisms to translate shared priorities into action. They reflect a collective vision of the CKD Clinic Network as a learning-oriented, patient-centred platform capable of driving system-level improvement.

## Limitations

This work should be interpreted in light of several limitations. The survey response rate was approximately 24%, which may limit the generalizability of findings and introduces the potential for response bias. Some attrition occurred as respondents completed the survey. This partial completion trend is not uncommon in online surveys of this length and complexity and may reflect factors such as respondent burden or relevance of later items. In addition, the survey was conducted over a relatively short 10-day period to ensure results were available in advance of the fixed timing of the World Café session, which may have further constrained participation.

Participation in the World Café was based on purposive sampling from individuals who had previously expressed interest in CKD Clinic Network activities. While this approach enabled inclusion of diverse roles and perspectives, it may have resulted in selection bias toward more engaged members of the community. Geographic representation was also limited, with participants representing 6 of 13 provinces and territories; perspectives from Prince Edward Island, Newfoundland and Labrador, New Brunswick, Saskatchewan, and the Territories were not captured.

Although patient partners were included, their representation was limited, with three completing the survey and two participating in the World Café. As such, findings may not fully reflect the breadth and diversity of patient perspectives. Finally, this work reflects a single point in time; priorities and perspectives may evolve as the CKD landscape, including emerging therapies and models of care, continues to change.

Taken together, the survey findings and World Café outputs are best interpreted as contextual, exploratory insights intended to inform ongoing dialogue and future work, rather than definitive or consensus-based priorities.

## Implications

The findings from this World Café align with the growing emphasis on Learning Health Systems (LHS) as a model for integrating research, data, and clinical practice to drive continuous improvement in health outcomes. LHS approaches emphasize real-time use of evidence, embedded research within care delivery, and iterative feedback loops that inform decision-making. The themes identified in this work reflect core components of LHS frameworks and suggest a readiness within the CKD community to move toward more integrated, learning-oriented models of care.

More broadly, this work contributes to an evolving body of literature on network-based and participatory approaches to health system transformation. Notably, unlike traditional priority-setting exercises that focus on consensus-driven ranking, the World Café methodology enabled open, generative dialogue and surfaced a wider range of perspectives and possibilities. The findings also align with national CKD strategies that emphasize equitable access to care, integration of patient-reported outcomes, and the adoption of new therapies, while underscoring ongoing challenges related to variability in care delivery and system-level coordination.

Together, the survey and World Café conversations reflect a shared aspiration to evolve the CKD Clinic Network from a knowledge-sharing platform into a driver of coordinated research and practice-based change. This transformation points toward a more formalized, nimble, and equity-oriented community of practice that is actively engaged in identifying shared priorities and integrating new knowledge into care across diverse settings.

Next steps include co-developing a refreshed vision and a supporting value proposition with network members to clearly articulate the purpose and future role of the network. A public-facing website will serve as a virtual hub, offering tools, links to events, and opportunities for peer learning and connection. In piloting the repositioning of the network, early efforts will focus on defining common objectives, advancing local-to-national collaboration, and building sustainable governance and funding models. These efforts will be grounded in partnerships with research, policy, and patient communities, and intentionally aligned with existing national platforms to amplify impact and reach.

## Supplemental Material

Supplemental Material - Repositioning the Canadian CKD Clinic Network Through Structured Input and World Café Dialogue: A Conference Report Supplemental Material for Repositioning the Canadian CKD Clinic Network Through Structured Input and World Café Dialogue: A Conference Report by Selina Allu, Mila Tang, Monica C. Beaulieu, Aminu K. Bello, Micheli Bevilacqua, Helen Hoi-Lun Chiu, Kate Chong, Maoliosa Donald, Mark D Elliott, Meghan J Elliott, Manuel Escoto, Isabella Ethier, Janet Graham, Adeera Levin, Judith G Marin, Kelly Picard, Steven D Soroka, Christine A White, Cathy Woods, Tae Won Yi, Matthew T. James in Canadian Journal of Kidney Health and Disease

## ORCID iDs

Selina Allu https://orcid.org/0000-0003-4084-2491

Mila Tang https://orcid.org/0000-0003-3459-5826

Monica C. Beaulieu https://orcid.org/0000-0003-2023-4317

Aminu K. Bello https://orcid.org/0000-0002-6905-5937

Micheli Bevilacqua https://orcid.org/0000-0001-8321-7413

Helen Hoi-Lun Chiu https://orcid.org/0000-0002-7685-8324

Maoliosa Donald https://orcid.org/0000-0002-8484-8361

Meghan J Elliott https://orcid.org/0000-0002-5434-2917

Steven D Soroka https://orcid.org/0000-0002-7278-1702

Tae Won Yi https://orcid.org/0000-0003-3350-1170

Matthew T. James https://orcid.org/0000-0002-1876-3917
