# Supplementary material for: Repositioning the Canadian CKD Clinic Network Through Structured Input and World Café Dialogue: A Conference Report
Source: Can J Kidney Health Dis. 2026 Jul 17;13:20543581261470157. doi: 10.1177/20543581261470157 (PMC13379674; doi:10.1177/20543581261470157)
Supplement: Supplemental Material - Repositioning the Canadian CKD Clinic Network Through Structured Input and World Café Dialogue: A Conference Report [file sj-pdf-1-cjk-10.1177_20543581261470157.pdf]

CJKHD-26-0038.R3

Repositioning the Canadian CKD Clinic Network Through Structured Input and World Café Dialogue: A Conference Report

Résumé

Objectif du programme

Ce rapport de conférence présente les conclusions d’activités destinées à repositionner le Réseau canadien des cliniques d’insuffisance rénale chronique (IRC) en cernant des priorités de recherche et des actions à mettre en œuvre conformément aux lignes directrices de 2024 de KDIGO (Kidney Disease: Improving Global Outcomes). L’initiative visait à définir des mesures prioritaires pour intégrer la recherche clinique et les soins, au moyen d’un dialogue collaboratif et d’une planification guidée par les besoins de la communauté.

Sources de l’information

Les lignes directrices de pratique clinique KDIGO de 2024 pour l’IRC ont servi de cadre pour définir et hiérarchiser les priorités de recherche. Les informations ont été obtenues au moyen d’un sondage national et d’un atelier participatif utilisant la méthodologie *World Café*.

Méthodologie

Un sondage en ligne anonyme et semi-structuré a été mené auprès des membres du Réseau canadien des cliniques d’IRC du 4 au 14 avril 2025 afin d’évaluer la pertinence et l’importance perçues des recommandations de recherche des lignes directrices KDIGO de 2024. Le sondage a été suivi, en mai 2025, d’un atelier *World Café* en présentiel réunissant des patients, des cliniciens, des chercheurs et des gestionnaires. Les données, constituées de représentations graphiques réalisées en direct (*graphic recording*), de notes de table et de commentaires consignés sur des notes autocollantes, ont été soumises à une analyse thématique.

Principaux résultats

En tout 80 membres du réseau ont répondu au sondage (taux de réponse : 24 %). Plusieurs priorités de recherche ont été dégagées : effet des nouveaux traitements (iSGLT2, antagonistes non stéroïdiens des récepteurs des minéralocorticoïdes) chez les patients intolérants aux iECA/ARA; recherche en science de la mise en œuvre visant à soutenir l’intégration des traitements éprouvés et de la gestion des symptômes; cliniques de transition pour les jeunes;

outils de littératie en santé; prévention de l'hyperkaliémie; déprescription médicamenteuse; effets des restrictions alimentaires; et identification, classification et contrôle des symptômes.

Les participants à l'atelier *World Café* (n=19) ont relevé plusieurs forces du réseau, notamment des relations bien établies favorisant la collaboration à l'échelle nationale, une culture de partage des connaissances et l'intégration de la perspective des patients. La rétroaction a mis l'accent sur la nécessité d'une mise en œuvre collaborative et d'une application contextuelle des lignes directrices. L'atelier a également permis de déterminer l'orientation future du réseau en se basant sur les principes d'un système de santé apprenant, lequel intègre la recherche aux soins courants et favorise l'alignement avec les stratégies provinciales et nationales en IRC, la participation des patients, l'utilisation d'outils de mesure communs et des boucles d'apprentissage locales et nationales. Les principales mesures retenues visaient à accroître la visibilité du réseau grâce à un site Web public, à arrimer ses activités à des plateformes nationales durables et à favoriser le développement de nouveaux partenariats et de collaborations en recherche.

Limites

Le faible taux de réponse au sondage (24 %) et de participation à l'atelier *World Café* (19 membres) limite les résultats aux perspectives d'une faible proportion du réseau. Par conséquent, les résultats pourraient omettre certains défis ou occasions importantes qui n'ont pas émergé en raison de l'absence des perspectives de l'ensemble de la communauté des soins rénaux.

Conclusion

Cette initiative démontre que la prise en compte de perspectives diversifiées peut guider la transformation du réseau et les orientations stratégiques futures de la recherche. Le Réseau des cliniques d'IRC est bien positionné pour évoluer vers une plateforme réactive et centrée sur le patient, et favoriser l'apprentissage et l'amélioration continue de la prestation des soins rénaux au Canada.
